# Sacral terminal filar cyst: a distinct variant of spinal meningeal cyst and midterm clinical outcome following combination resection surgery

**DOI:** 10.3389/fsurg.2023.1272580

**Published:** 2023-11-03

**Authors:** Guozhong Lin, Chenlong Yang, Tao Yu, Jia Zhang, Yu Si, Chao Wu, Changcheng Ma, Bin Liu, Jun Yang, Jingcheng Xie

**Affiliations:** Department of Neurosurgery, Peking University Third Hospital, Beijing, China

**Keywords:** spinal meningeal cyst, sacral terminal filar cyst, filum terminale, MRI, surgery, outcome

## Abstract

**Objective:**

Spinal meningeal cysts (SMCs) are currently classified into three types: extradural cysts without nerve root fibers (Type I), extradural cysts with nerve root fibers (Type II), and intradural cysts (Type III). However, the sacral terminal filar cyst is a distinct subtype with the filum terminale rather than nerve roots within the cyst. This study aimed to investigate the clinicoradiological characteristics and surgical outcomes of sacral terminal filar cysts.

**Methods:**

A total of 32 patients with sacral terminal filar cysts were enrolled. Clinical and radiological profiles were collected. All patients were surgically treated, and preoperative and follow-up neurological functions were evaluated.

**Results:**

Chronic lumbosacral pain and sphincter dysfunctions were the most common symptoms. On MRI, the filum terminale could be identified within the cyst in all cases, and low-lying conus medullaris was found in 23 (71.9%) cases. The filum terminale was dissociated and cut off in all cases, and the cyst wall was completely resected in 23 (71.9%) cases. After a median follow-up period of 26.5 ± 15.5 months, the pain and sphincter dysfunctions were significantly improved (both *P *< 0.0001). The cyst recurrence was noted in only 1 (3.1%) case.

**Conclusions:**

Sacral terminal filar cysts are rare, representing a distinct variant of SMCs. Typical MRI features, including filum terminale within the cyst and low-lying conus medullaris, may suggest the diagnosis. Although the optimal surgical strategy remains unclear, we recommend a combination of resection of the cyst wall and dissociation of the filum terminale. The clinical outcomes can be favorable.

## Introduction

Spinal meningeal cysts (SMCs) refer to extradural cystic lesions communicating with the subarachnoid space via a focal dural defect (1, 2). SMCs of the sacral region, also known as sacral cysts, are relatively common findings in patients being evaluated for low back radicular pain. Sacral cysts are generally considered to be congenital lesions (3). The definite incidence of SMCs remains unclear; nevertheless, according to previous reports, the prevalence of sacral cysts can reach up to 17% in patients undergoing myelography for the investigation of sciatica (4). The most widely accepted hypothesis regarding the pathogenesis of SMCs is that cerebrospinal fluid herniates through a weak part of the spinal dura, and this herniation often forms a one-way valve. Cerebrospinal fluid accumulates into the cyst and fails to flow out, enlarging the cyst gradually and causing spinal cord or nerve root compression symptoms. Nabors and colleagues proposed a classification, in which SMCs are divided into three categories: Type I, extradural SMCs without spinal nerve root fibers; Type II, extradural SMCs with spinal nerve root fibers; and Type III, intradural SMCs (5). Additionally, Type I is further divided into two subgroups: Type IA, extradural meningeal cyst or extradural arachnoid cyst; and Type IB, meningeal cysts in the sacral canal (mostly located in S1-S3). Type II is also known as Tarlov's perineural cyst or a spinal nerve root diverticulum, and Type III actually refers to a spinal intradural arachnoid cyst (5). However, SMCs occurring in the filum terminale is a distinct subtype, as this extremely rare entity has filum terminale rather than nerve root within the cyst, and we named it as sacral terminal filar cyst (6). Due to the relative rarity, the diagnosis and treatment of this special subtype have not yet been fully understood. This study aimed to clarify the clinicoradiological characteristics, surgical strategies, and outcomes of sacral terminal filar cysts.

## Materials and methods

### Patients

This retrospective study has been approved by the Institutional Review Board and Ethics Committee of Peking University Third Hospital. A total of 32 patients with sacral terminal filar cyst were enrolled, including 13 males and 19 females. The average age was 36.1 ± 11.7 years, ranging from 16 to 64 years. The duration of symptoms prior to surgery was 30.2 ± 25.9 months, ranging from 10 days to 10.5 years.

Inclusion criteria included: (1) A definitive diagnosis of sacral terminal filar cyst based on spinal MRI; (2) symptomatic sacral cyst manifesting as pain, lower-extremity weakness, and/or sphincter disturbance; (3) the sacral cyst was surgically treated, and filum terminale was found within the cyst intraoperatively; and (4) complete follow-up data including clinical symptoms, physical examinations, and radiological imaging. Exclusion criteria were as follows: (1) accompanied other type SMCs; (2) concomitant other spinal diseases; or (3) a previous history of operation on the SMCs.

### Clinicoradiological evaluation

The pain intensity was evaluated as per the Visual Analogue Scale (VAS). Sphincter dysfunctions were assessed using the Japanese Orthopaedic Association (JOA) scoring system: 0 point represents urodialysis or urinary incontinent; 1 point, sense of retention, difficulty with micurature, prolonged urination, or dysuria; 2 points, delayed urination, or frequent urination; 3 points represent normal functions (7). The JOA score for the lumbar spine was used to evaluate neurological status. The JOA improvement index and improvement rate were calculated according to the following formula: improvement index = postoperative JOA score—preoperative JOA score; improvement rate = [(postoperative JOA score—preoperative JOA score)/(29—preoperative JOA score)] × 100%. Perioperative and follow-up spinal MRIs were available for all patients, which were used to evaluate the size of the cyst, the level of the conus medullaris, the recovery of the tethered cord, and postoperative recurrence of cysts.

### Surgical strategies

**Figure 1:**
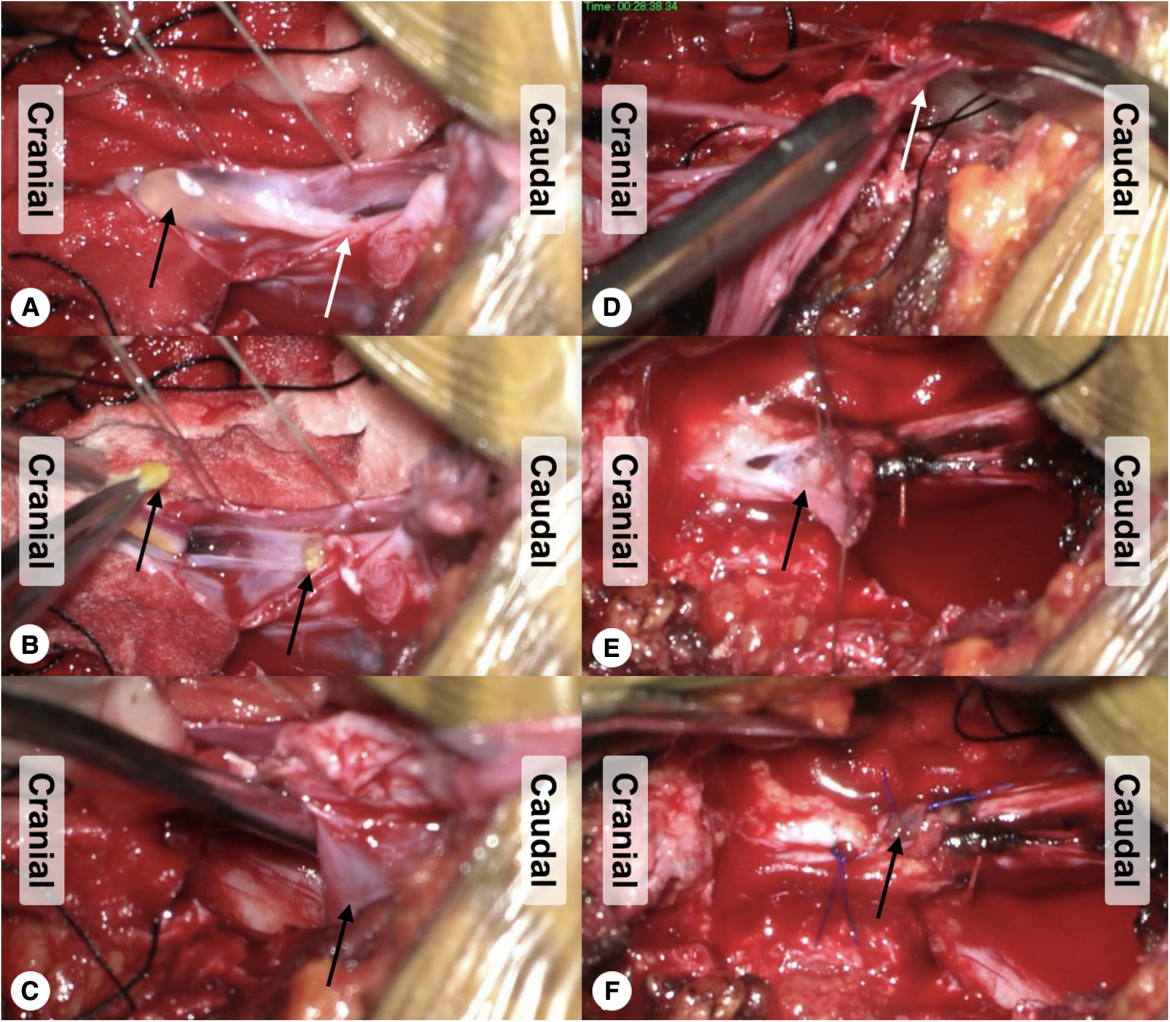
Surgical procedures and intraoperative findings. (**A**) After incision of the cyst and dorsal wall of the dural sac, a thickened filum terminale with fatty infiltration was noted (black arrow), and the cerebrospinal fluid flows into the cyst through the orificium (white arrow). (**B**) The caudal end of the intradural filum terminale was cut off by bipolar electrocoagulation (black arrows indicating the broken ends of the filum terminale). (**C**) The cyst wall (black arrow) was dissociated from the surrounding adhesion bluntly. (**D**) The dissociated cyst wall was resected with the filum terminale externum (white arrow). (**E**) The caudal end of the dural sac was dissociated and tightly sutured (black arrow). (**F**) The dural mater was tightly closed, and the terminal cistern was reconstructed (black arrow).

Combined intravenous and inhalation anesthesia, as well as intraoperative neurophysiological monitoring, was routinely performed. The patient was placed in a prone position with the lumbosacral portion at the highest level. A laminotomy was performed in the posterior wall of the sacral canal, following which the cyst was exposed. Next, the adhesion between the cyst and the inner wall of the spinal canal was dissected under a microscope. Then, the cyst was incised dorsally and unfolded, and the filum terminale was found passing through the orificium in the caudal end of the dural sac. From the orificium, cerebrospinal fluid constantly flowed out. To expose the filum terminale internum in the dural sac and to identify the orificium where the filum terminale internum breaks through the dural sac, a posterior midline incision of the dorsal dura was made. Subsequently, the caudal portion of the filum terminale internum was cut off, and a small part of the surrounding dural sac was resected. The cyst wall and the filum terminale externum were also dissociated and resected (Figure 1). During this procedure, the adhesion and the tethered cord were thoroughly released till the cauda equina was eased completely. At last, the caudal end of the dural sac was appropriately dissociated and tightly sutured, and the terminal cistern was reconstructed. Main surgical procedure video is given in Supplementary Video 1.

### Statistical analysis

GraphPad Prism 9.3.1 (GraphPad, San Diego, CA, USA) was used for statistical analyses. The normality of data was examined using the Kolmogorov–Smirnov test. Continuous variables were presented as “mean ± standard deviation (SD)” when normally distributed or “medians (interquartile ranges, IQR)” when non-normally distributed. Categorical variables were presented as percentages. Statistical comparisons were performed using Mann–Whitney *U*-test or Student *t*-test for continuous variables, as appropriate. The threshold for significance was set as a *P* value less than 0.05.

## Results

### Clinical manifestations

Among the 32 patients, 24 (75.0%) presented with chronic lumbosacral or perineal pain, among whom 13 had radiating pain spreading along the affected nerve root. Additionally, other onset symptoms included numbness in the lower extremities (15/32; 46.9%), weakness in the lower extremities (18/32; 56.3%), and sphincter dysfunctions (17/32; 53.1%).

Physical examinations revealed that 11 (34.4%) patients had decreased acupuncture sensation at the saddle area, 18 (56.3%) patients had decreased muscle strength in the gastrocnemius, 3 (9.4%) patients had muscle atrophy, and 25 (78.1%) patients had weakened Achilles tendon reflex. No pathological reflection of Babinski's sign was induced. The VAS scores ranged from 0 to 7 points (mean 3.9 ± 2.5 points). The mean JOA score for sphincter dysfunctions was −1.8 ± 1.8 points (range, −6–0 points). The mean JOA score for the lumbar neurological functions was 17.0 ± 7.9 points (range, 5–28 points).

### Radiological features

**Figure 2:**
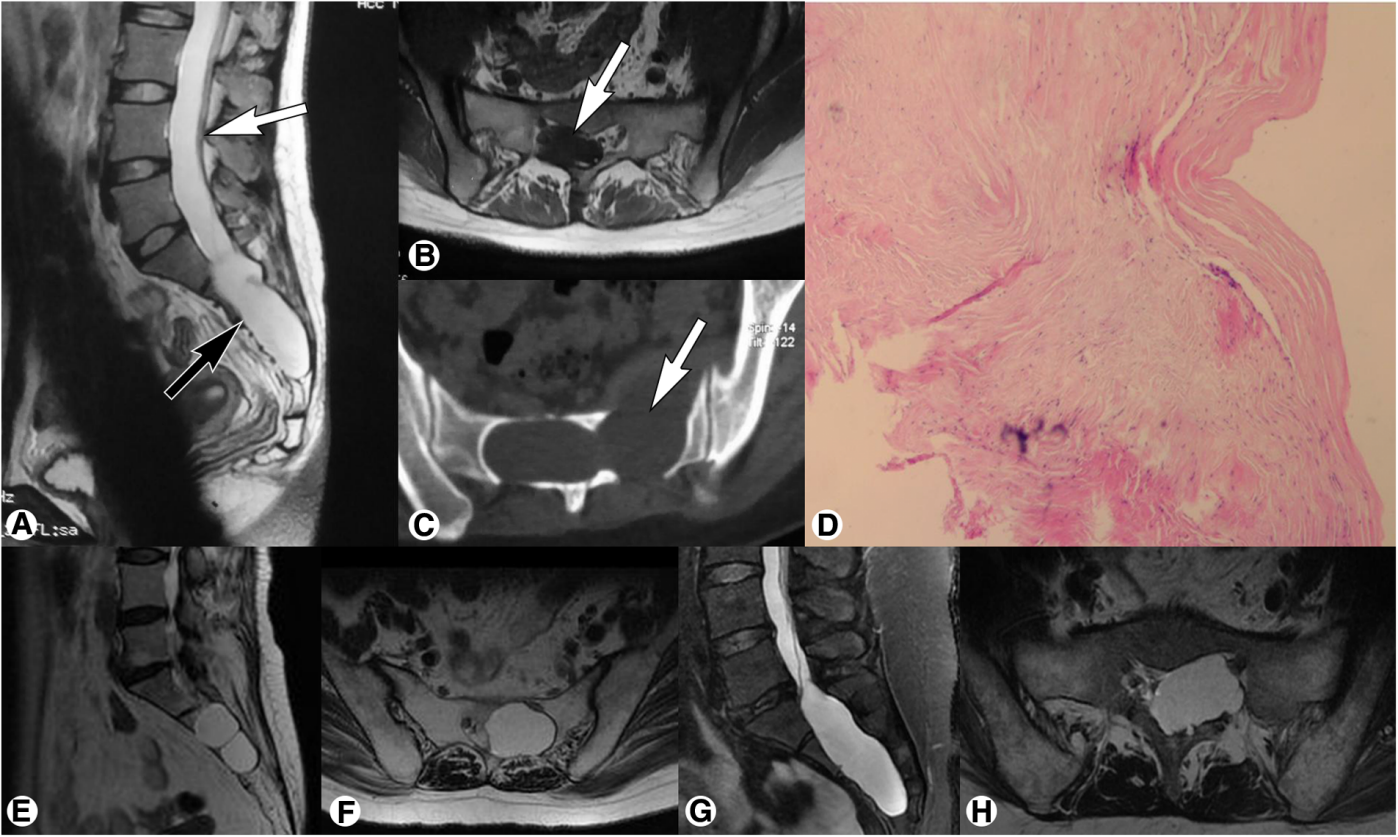
Preoperative magnetic resonance imaging of sacral terminal filar cyst. (**A**) Spinal sagittal T2-weighted imaging showed a sacral cyst (black arrow) accompanied by low-lying conus medullaris at the inferior margin of the L4 vertebral body (white arrow). (**B**) Axial T1-weighted imaging demonstrated a thickened filum terminale with fatty infiltration (white arrow) within the cyst. (**C**) Axial computed tomography showed local bone destruction (white arrow) at the anterior and posterior walls of the sacral canal. (**D**) Histopathological examination showed fibrous connective tissue with a cyst wall-like structure, which was consistent with the diagnosis of meningeal cysts. (**E,F**) Representative magnetic resonance imaging of Nabors Type IB meningeal cyst showed an extradural cyst without spinal nerve root fibers (**E**, sagittal T2-weighted; **F**, axial T2-weighted). (**G,H**) Representative magnetic resonance imaging of Nabors Type II meningeal cyst showed an extradural cyst with spinal nerve root fibers (**G**, sagittal T2-weighted; **H**, axial T2-weighted).

Spinal MRI demonstrated an isolated cystic lesion in the sacral canal in all cases. The locations included: L5-S2 levels in 4 cases, S1-S3 levels in 11 cases, S2-S3 levels in 9 cases, S2-S4 levels in 6 cases, and S1-S4 levels in 2 cases. The cystic lesions showed hypointensity on T1-weighted imaging and hyperintensity on T2-weighted imaging; no enhancement was observed after the administration of contrast medium. The filum terminale was identified within the cyst in all (100%) cases, which was thickened with fatty infiltration, and low-lying conus medullaris was found in 23 (71.9%) cases (Figure 2).

### Perioperative findings

The operations lasted for 1.5–3 h (mean 1.6 ± 0.3 h). Intraoperative blood loss ranged from 30 ml to 250 ml (mean 76.3 ± 43.8 ml). The filum terminale was dissociated and cut off in all (100%) cases, and the cyst wall was completely resected in 23 (71.9%) cases and subtotally resected in 9 (28.1%) cases. The postoperative course was uneventful in all cases, and there were no significant motor complications or sphincter deterioration. Only 8 patients reported mild perianal numbness, which was gradually recovered within 3 months.

All resected specimens were subjected to pathological examinations. The cyst wall showed fibrous connective tissue with a cyst wall-like structure, and a small part of it was lined with squamous epithelium, which was consistent with the diagnosis of meningeal cysts. The resected filum terminale was thickened with fibrous tissue and adipose hyperplasia.

### Clinical outcomes

The follow-up period ranged from 12 months to 7 years (mean, 26.5 ± 15.5 months). The pain, sensorimotor disturbances, and sphincter dysfunctions were all significantly improved. The postoperative mean VAS score was significantly lower than that before surgery (1.4 ± 1.2 vs. 3.9 ± 2.5; *t *= 7.673; *P *< 0.0001). The postoperative mean JOA score for sphincter dysfunctions was −0.28 ± 0.89, which was significantly better than the preoperative score (*t *= 4.980; *P *< 0.0001). The postoperative mean JOA score for lumbar neurological functions was 24.9 ± 3.0, which was significantly higher than the preoperative score (*t *= 8.506; *P *< 0.0001). The JOA improvement index was 9.4 ± 6.6 (range 1.0–20.0), and the improvement rate was 68% ± 13% (range 33%–100%). The detailed neurological function assessment results are summarized in Table 1. According to the follow-up MRI, the cyst recurrence was only noted in one (3.1%) case. The spinal cord tethering was completely relieved in all cases.

**Table 1:** Clinical characteristics and neurological functions of patients.

Case No.	Gender/age (years)	Duration (months)	Maximal diameter (cm)	Follow-up time (months)	Preop VAS score	Postop VAS score	Preop JOA sphincter score	Postop JOA sphincter score	Preop JOA lumbar score	Postop JOA lumbar score	Preop JOA total score	Postop JOA total score	Improvement index	Improvement rate
1	M/16	0.33	2.10	18	4	2	0	0	22	26	22	26	4	0.57
2	F/34	3.00	4.10	18	7	3	−3	0	8	22	5	22	17	0.71
3	F/37	6.00	2.90	27	6	1	−3	0	10	24	7	24	17	0.77
4	M/60	126.00	3.00	24	0	0	0	0	28	29	28	29	1	1.00
5	F/54	4.50	2.50	24	5	1	−3	0	18	26	15	26	11	0.79
6	M/42	3.00	2.60	30	4	3	0	0	21	27	21	27	6	0.75
7	F/39	12.00	5.10	36	6	1	−3	0	8	21	5	21	16	0.67
8	F/38	22.00	2.80	30	5	3	−3	0	10	24	7	24	17	0.77
9	F/52	24.00	3.50	42	6	3	−3	0	9	22	6	22	16	0.70
10	M/37	36.00	5.50	36	0	0	0	0	27	28	27	28	1	0.50
11	F/34	42.00	3.10	24	7	3	−3	0	6	20	3	20	17	0.65
12	F/25	30.00	2.40	15	0	0	0	0	26	28	26	28	2	0.67
13	M/21	24.00	2.90	15	0	0	0	0	27	29	27	29	2	1.00
14	F/19	48.00	2.80	24	0	0	0	0	26	28	26	28	2	0.67
15	F/64	24.00	3.10	84	4	0	−3	0	19	26	16	26	10	0.77
16	M/28	24.00	2.80	60	0	0	0	0	26	28	26	28	2	0.67
17	F/32	15.00	3.90	24	4	3	0	0	22	27	22	27	5	0.71
18	F/35	42.00	4.30	42	6	3	−3	0	10	25	7	25	18	0.82
19	M/34	24.00	2.50	48	4	2	0	0	23	26	23	26	3	0.50
20	F/44	66.00	3.90	27	6	3	−3	0	10	24	7	24	17	0.77
21	F/37	72.00	3.80	24	5	1	−3	0	11	25	8	25	17	0.81
22	M/42	60.00	3.10	24	6	1	−6	0	5	19	−1	19	20	0.67
23	F/28	36.00	2.90	12	0	0	0	0	26	28	26	28	2	0.67
24	M/30	15.00	2.20	18	4	3	0	0	24	27	24	27	3	0.60
25	F/37	42.00	4.70	18	0	0	0	0	26	27	26	27	1	0.33
26	M/35	24.00	2.70	12	6	1	−3	−3	10	21	7	18	11	0.50
27	F/26	60.00	2.30	12	6	3	−3	0	9	19	6	19	13	0.57
28	F/26	30.00	3.60	24	4	1	0	0	23	27	23	27	4	0.67
29	M/17	12.00	2.00	15	5	1	−3	−3	12	24	9	21	12	0.60
30	F/46	8.00	2.50	12	6	2	0	0	14	25	14	25	11	0.73
31	M/52	15.00	2.80	12	4	0	−3	0	22	26	19	26	7	0.70
32	M/33	16.00	3.20	18	6	1	−6	−3	6	20	0	17	17	0.59

M, male; F, female; Preop, preoperative; Postop, postoperative; VAS, visual analogue scale; JOA, Japanese orthopedic association scores.

## Discussion

The most common type of SMCs is Tarlov cyst (Nabors Type II) with nerve roots (2, 8), followed by sacral cyst (Nabors Type IB) without any structure inside (9). In this study, we reported a distinct subtype of sacral cysts with filum terminale inside. Sun et al. considered it as a special variant of Nabors Type I cyst (9). However, in the current study, we found that clinical symptoms, imaging features, and surgical outcomes of sacral terminal filar cysts were quite unique and different from those of conventional Nabors Type I cysts. Therefore, we recommend that it should be classified separately as a distinct subtype of sacral cysts: sacral terminal filar cyst (6).

The etiology and pathogenesis of sacral terminal filar cysts remain unclear. We found that the neck of cysts was mostly located at the site where the filum terminale breaks through the dural sac, and thus we speculated that the focal weakness of the dura mater may be the anatomical basis for the occurrence of sacral terminal filar cysts. Generally, the filum terminale internum transitions into the filum terminale externum at the caudal end of the dural sac, accordingly the focal dura mater in this site is similar to the nerve root sleeve. Therefore, we consider that sacral terminal filar cysts may share a similar pathogenetic mechanism with sacral Tarlov cysts.

Clinically, sacral terminal filar cysts are relatively rare. With the enlargement of the sacral terminal filar cyst and progressively increasing intracystic pressure, the cyst may compress the surrounding structures and causes pain in the perineum (10, 11). Additionally, sacral terminal filar cysts are often associated with tethered spinal cord symptoms such as chronic lower back pain, lower-extremity weakness and muscle atrophy, and dysfunctions of the bladder and bowel. Accompanying tethered spinal cord can help clinicians to distinguish sacral terminal filar cysts from other sacral cyst subtypes (6, 12).

MRI is the preferred imaging modality for the diagnosis of sacral cysts (13, 14). The radiological features of sacral terminal filar cysts are similar to those of other sacral cyst subtypes, showing cystic lesions without contrast enhancement (14, 15). Noteworthily, the filum terminale can be visible within the sacral terminal filar cyst, and low-lying conus medullaris may be present (6, 16). The filum terminale can be easily identified due to fatty infiltration, manifesting as linear fat strip signals. The filum terminale without fatty infiltration may be confused with nerve roots (17). However, the filum terminale is generally thicker and can be traced from the end of the spinal cord proceeding gradually downward to the cyst continuously.

Sacral nerve root irritation and tethered spinal cord symptoms are indications of surgical treatment of sacral terminal filar cysts (18). The surgical goals and strategies for treating sacral terminal filar cysts are distinct from those for other sacral cyst subtypes (2, 19). Considering simply cutting off the filum terminale externum cannot relieve the spinal cord tethering sufficiently, we chose to cut off the filum terminale internum in the dural sac. Moreover, simply ligating the cyst neck at the orificium fistulae without removing the filum terminale would not completely seal the cerebrospinal fluid leakage. Intraoperatively, we cut off the filum terminale subdurally and resected a small part of the surrounding dural sac. To locate the orificium fistulae, we need to incise the dural sac along the dorsal midline, and the weak part of the dura mater was exposed where the filum terminale break through the dural sac. Then, we dissociated the filum terminale internum, and the tethered spinal cord was completely relieved (20). Additionally, the caudal end of the dural sac was appropriately dissociated and tightly sutured, and the terminal cistern was reconstructed.

Surgical outcomes of sacral terminal filar cysts are satisfactory. Neurological deficits were all recovered, and cyst recurrence was only noted in one case after a mean follow-up period of 26.5 ± 15.5 months. Therefore, we propose that surgical treatment combining resection of the cyst wall and releasing the tethered spinal cord by dissociation of the filum terminale is a safe and efficient approach leading to a favorable prognosis. Furthermore, reconstruction of the terminal cistern is a crucial procedure for eliminating cerebrospinal fluid leakage and reducing the probability of cyst recurrence.

## Conclusions

Sacral terminal filar cysts represent a distinct subtype of SMCs, with typical MRI characteristics including filum terminale within the cyst and low-lying conus medullaris. A combination of resection of the cyst wall and dissociation of the filum terminale is a safe and effective surgical approach, and the clinical outcomes are favorable.

## Data Availability

The original contributions presented in the study are included in the article/**Supplementary Material**, further inquiries can be directed to the corresponding authors.
